# The Use of Piezoelectric Osteotomy Instruments During a Rhinoplasty Operation in a Patient With a Cochlear Implant

**DOI:** 10.7759/cureus.44833

**Published:** 2023-09-07

**Authors:** Cagri Kulekci

**Affiliations:** 1 Otolaryngology, Hacettepe University Faculty of Medicine, Ankara, TUR

**Keywords:** device failure, hearing loss, rhinoplasty, piezosurgery, cochlear implant

## Abstract

A cochlear implant is a life-changing operation that significantly improves the lives of patients. Those with cochlear implants require ongoing measures to ensure the longevity of both their devices and their well-being. One concerning issue is the potential device failure due to the use of surgical instruments during procedures. In this report, we present a successful case of utilizing an ultrasonic osteotome for a primary septorhinoplasty in a 22-year-old patient who had undergone cochlear implant surgery 17 years prior. To our knowledge, this is the first recorded instance of applying a piezoelectric tool on a patient with a cochlear implant. Our findings support the safety of using a piezo osteotome in cochlear implant recipients.

## Introduction

Cochlear implant surgery is an established and successful treatment with historical backgrounds reaching as far back as the 1960s. One of the most common indications for cochlear implantation surgery is congenital profound sensorineural hearing loss. Cochlear implantation restores the hearing capacity of the individual, removing a barrier to achieving their full potential such as receiving a higher education. Considering the early ages the patients are operated on, their wishes and needs grow as they grow older like any other human being. As cosmetic procedures are becoming ever more popular in younger generations [1], the number of cosmetic procedures performed in patients with cochlear implantation is expected to increase. Here in this paper, we would like to present a patient with a cochlear implant who wishes to undergo a septorhinoplasty operation and a first case report of a rhinoplasty operation undertaken using piezosurgical osteotomes.

## Case presentation

**Figure 1:**
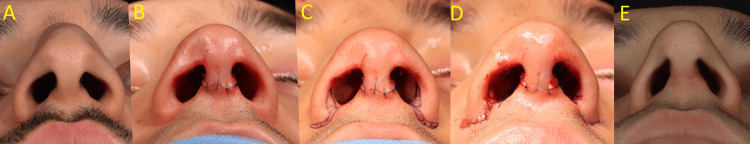
Photographs from the basal view. (A) preoperative, (B) intraoperative without alar base reduction markings, (C) intraoperative with markings, (D) immediate postoperative, (E) post-op six months

A 22-year-old male presented to the clinic with breathing problems and concerns about the external appearance of his nose. He had undergone a right cochlear implantation surgery for bilateral profound hearing loss at our clinic when he was five years old and was otherwise healthy. He has developed the necessary language and speech abilities and he is now attending a university as an engineering major. Physical examination revealed a left-sided septal deviation, a large nasal hump, and a droopy nasal tip with large and flared nostrils. A septorhinoplasty was planned. Preoperative photodocumentation was performed and post-op design was discussed with the patient and decided upon. Because of the ease and precision of bone works like chiseling, osseoprofiling, and osteotomies, the author uses piezosurgical instruments. The patient was informed about the piezo technology and the surgeon’s use of it, and he was also informed that piezo was intended to be used in his operation. After the initial interview, a literature search was performed to confirm the safety of piezosurgery to be performed in patients with cochlear implants. The search using “cochlear implantation, piezosurgery” resulted in five papers in the English language. Three of the five articles dealt with piezoelectric stimulation of the ear, and two of them were about the drilling of the temporal bone. No information was available about the safety of using piezosurgical instruments on a cochlear implant patient. Because piezosurgery uses ultrasound waves and specifically works on bones and no electric current is produced during its performance, no complications were foreseen. However, a thorough search was performed about the principles of piezosurgery, and it was found to be safe and eligible for the patient's operation. All the information was communicated and discussed with the patient, and he consented to piezo being used in his operation. Primary septorhinoplasty was performed under general anesthesia. Following oral intubation, the patient was prepped and draped in the usual fashion. A reverse V incision was placed on the columellar skin and cartilages, and a bony vault was elevated in the subperichondrial and subperiosteal plane. Elevation was performed high in the glabellar region and laterally as the inferior orbital rim and pyriform aperture. Septal elevation was performed bilaterally, and hump resection was performed conventionally with a Rubin osteotome. The resultant open vault and nasal bones were rasped and shaped with various piezo tips. Lateral nasal walls were thinned, and a nasofacial groove was pronounced also with piezosurgical instruments. Lateral wall procedures are especially safe and efficient with piezo and make the subsequent osteotomies easier. Bilateral lateral and transverse osteotomies were performed also with piezo, and with a final fine rasping, a piezosurgical intervention was completed. The rest followed in a similar way: open vault closed, tip surgery finished, and incisions closed. As the last step in the surgery, alar base surgery ensued. The author’s modified L-shaped alar base and alar flare resection were performed (Figure 1), and internal and external splints were applied.

Following an uneventful extubation, the patient was transferred to the ward. As soon as he regained full consciousness, he was asked to wear the external piece to his cochlear implant. He could hear as well as he did before the surgery. Following a month, no problems or malfunctions were observed concerning his cochlear implant. He continued his therapy with the audiology department, and objective tests revealed no problems with the implanted electrode and the processor.

**Figure 2:**
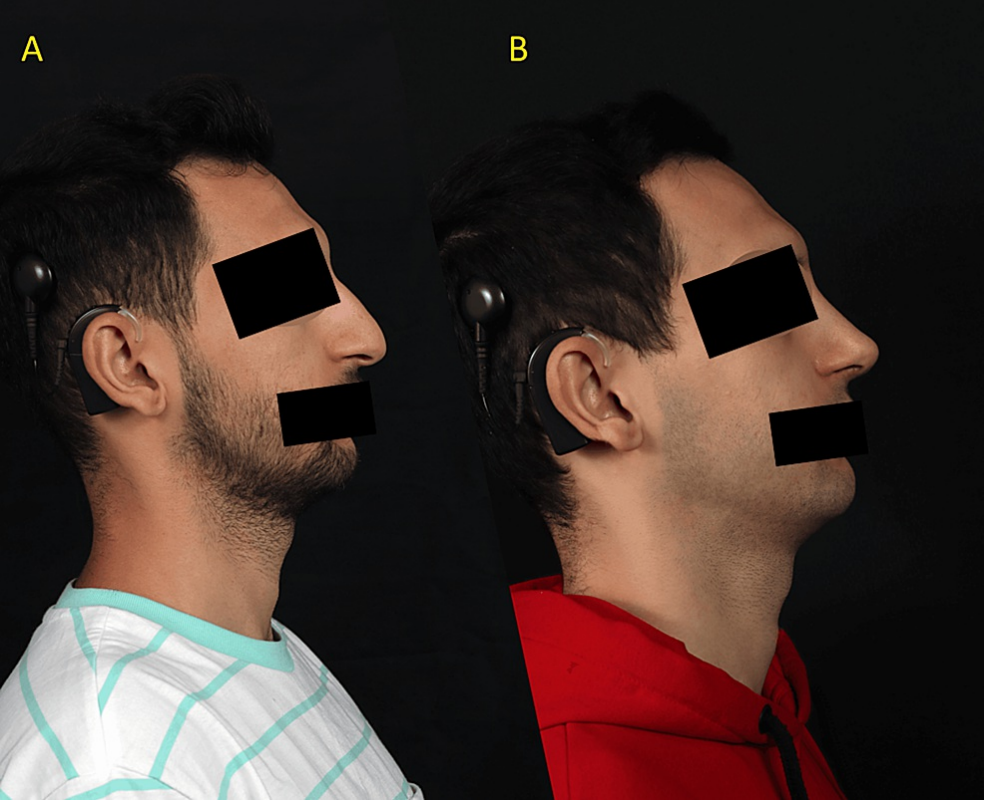
Right-side view of the patient. (A) preoperative side view and (B) postoperative six-month result. Note the cochlear implant in use

This is the first reported case in the English literature where a patient with a cochlear implant received piezosurgical instruments during the operation without any complications to the cochlear implant device. The preoperative side and six-month postoperative side view are demonstrated in Figure 2.

## Discussion

Cochlear implant surgery has been performed for more than 60 years in the world [2]. Cochlear implant surgery has been done for many years at our institution with nearly 3,000 patients both with normal and abnormal cochlear anatomies [3]. As these patients transition from infancy to adulthood, they are expected to lead normal and healthy lives. Living with a cochlear implant requires specific life-long measures and recommendations. Because surgical removal of the implant due to device failure is bothersome for the patient, and the revision is technically more difficult risking incomplete insertions due to cochlear fibrosis, utmost care should be given to maintain the functionality of the device. One of the key factors that may result in device malfunction is surgery. The use of a monopolar cautery may result in the current passing through the implanted electrode and cause excessive heating which may cause the processor to fail interfering with its electronic systems. Thus, bipolar cautery is indicated for surgery. Likewise, any surgical instrument that is meant to be used should be examined and studied before use in the surgical scenario.

The author uses piezosurgical instruments for almost all rhinoplasties and in selected cases of septal and turbinate surgeries. This information was given to the patient, and a careful study of the literature was undertaken. Prior reports have shown the safety of piezosurgical instruments in oral and maxillofacial, ENT, and neurosurgery operations [4-6], and some publications exist on its use in otologic surgery [7]. The literature favors piezo for its precision, less soft tissue disruption, and bone necrosis. Although the use of piezo in rhinoplasty has not been found to be detrimental to cochlear functions [8], no information regarding its use in patients with cochlear implants was found. This paper is the first reported case in the English literature and probably the first case in which piezotome is used successfully in a patient with a previous cochlear implantation. Although the patient currently benefits fully from the implant as he could before, further follow-up may reveal unforeseen device complications. Nonetheless, it is quite sufficient to say that piezosurgical instruments can safely be used in patients with cochlear implants.

## Conclusions

Piezosurgical instruments can be safely used for a variety of surgeries in patients with cochlear implants. Our one-year experience with a single case suggests a potential framework for future safety guidelines and prospective studies. The major limitation of this study is that it is based on a single case. As more cases are documented and technologies advance, a comprehensive understanding of the safety of this approach can evolve with time.

In conclusion, this case report marks a preliminary step toward establishing the compatibility of piezosurgery with cochlear implants. Further investigations involving diverse medical devices and larger cohorts are essential to solidify the procedure's safety and broaden its applicability.
